# Epithelial stem cells are formed by small-particles released from particle-producing cells

**DOI:** 10.1371/journal.pone.0173072

**Published:** 2017-03-02

**Authors:** Wuyi Kong, Xiao Ping Zhu, Xiu Juan Han, Mu Nuo, Hong Wang

**Affiliations:** Beijing Khasar Medical Technology Co. Ltd., Beijing, China; National University of Singapore, SINGAPORE

## Abstract

Recent spatiotemporal report demonstrated that epidermal stem cells have equal potential to divide or differentiate, with no asymmetric cell division observed. Therefore, how epithelial stem cells maintain lifelong stem-cell support still needs to be elucidated. In mouse blood and bone marrow, we found a group of large cells stained strongly for eosin and containing coiled-tubing-like structures. Many were tightly attached to each other to form large cellular clumps. After sectioning, these large cell-clumps were composed of not cells but numerous small particles, however with few small “naked” nuclei. The small particles were about 2 to 3 μm in diameter and stained dense red for eosin, so they may be rich in proteins. Besides the clumps composed of small particles, we identified clumps formed by fusion of the small particles and clumps of newly formed nucleated cells. These observations suggest that these small particles further fused and underwent cellularization. E-cadherin was expressed in particle-fusion areas, some “naked” nuclei and the newly formed nucleated cells, which suggests that these particles can form epithelial cells via fusion and nuclear remodeling. In addition, we observed similar-particle fusion before epithelial cellularization in mouse kidney ducts after kidney ischemia, which suggests that these particles can be released in the blood and carried to the target tissues for epithelial-cell regeneration. Oct4 and E-cadherin expressed in the cytoplasmic areas in cells that were rich in protein and mainly located in the center of the cellular clumps, suggesting that these newly formed cells have become tissue-specific epithelial stem cells. Our data provide evidence that these large particle-producing cells are the origin of epithelial stem cells. The epithelial stem cells are newly formed by particle fusion.

## Introduction

Epithelia are sheets of cells that constitute the lining of most organs of the body, such as the skin, gut, airway tracts, kidney ducts, liver, eyes and other glands. Among these regional different epithelia, the intestinal and skin epithelial layers are the most rapidly renewing tissues in the mammalian body [1, 2]. Therefore, epithelial stem cells that locate in these areas should have fast self-renewal activity for their entire life.

Unlike the bone marrow- and blood-derived stem cells, which are considered multipotent and are the source of life-long cell production [3, 4], epithelial stem cells are regional-specific. Use of different stem cell markers has revealed multiple niches for epidermal stem cells in the bulge area, basal layers, hair germ, and sebaceous gland [5–8]. The niches of epithelia stem cells are regional; examples are kidney papilla being the niche for kidney stem cells [9], intestinal crypt for intestinal stem cells [10], basal cells and parabronchial smooth muscle for lung epithelial stem cells [11, 12], and subepithelial layer of the colonic mucosa and immigrating bone-marrow–derived stem cells for colon stem cells [13, 14]. These reports provide evidence that these stem cells differentiate to only an epithelial lineage but not other cell lineages [15].

Most believe that basal epithelial stem cells need to self-renew to maintain life-long mature cell production, and the mechanism of postnatal stem-cell self-renewal is by asymmetric division [16, 17]. Asymmetric cell division is found in progenitor cell division that gives rise to two daughter cells with different developmental fates: one daughter will differentiate along a specific lineage, and the other has the potential to remain the stem cell identity and continue to divide in an asymmetric manner [17].

Although asymmetric cell division has been suggested as the mechanism of life-long cell supply, a recent report from imaging techniques in live mice indicated that dermal stem cells do not undergo asymmetric cell division during differentiation. Epidermal stem cells have equal potential to divide or directly differentiate [18]. Therefore, where the life-long cell supply comes from and which cell is the origin of epithelial stem cells remains unknown.

We have identified a group of large cells in mouse blood that contain numerous small particles. Multiple large cells connect to each other and differentiate into cellular clumps composed of small particles. Our data provide evidence that epithelial stem cells are newly made of small particles that are derived from these large cells in the blood. The particle-producing large cells are the origin of epithelial stem cells, and the morphology of these large cells has never been reported.

## Materials and methods

### Animals and materials

Balb/C and c57BL/6 mice were purchased from Vital River Laboratories (Beijing). GFP-transgenic mice (FVB.Cg-Tg(ACTB-EGFP)B5Nagy/J; Jackson Laboratory, Bar Harbor, ME, USA) were bred or maintained in the animal facility in Beijing Khasar Medical Technology. The mice received food and water *ad libitum*. The animal protocols were approved by the Beijing Khasar Medical Technology Animal Regulatory Office. All animal procedures followed the guidelines of the Animal Regulatory Office and relevant national and international guidelines. Octamer-binding transcription factor (Oct4), (sex-determining region Y)-box 2 (Sox2), E-cadherin, DNA antibodies were purchased from Santa Cruz Biotechnology (Santa Cruz, CA, USA). An anti-GFP antibody was obtained from Aves Lab (Tigard, OR, USA). Alexa Fluor-conjugated goat secondary antibodies were from Invitrogen (Carlsbad, CA, USA). The TUNEL assay kit was from Roche Life Science (Shanghai). Other chemicals were from Sigma Chemical (Shanghai).

### Preparation of fresh mouse blood fractions

Blood was collected from 8- to 10-week-old Balb/C, 8- to 10-week-old c57BL/6 or 8- to 16-week-old GFP mice via cardiac puncture under sterile conditions after CO_2_ euthanasia. Each syringe was prefilled with 100 μl heparin (0.5%) to prevent coagulation. The blood samples were then pooled, transferred to a 50-ml centrifuge tube, immediately diluted with 1x phosphate-buffered saline (PBS) at a ratio of 1:5 (blood:PBS), then centrifuged at 200×*g* for 5 min. After removing the supernatant, the cellular portion was diluted with PBS at a 1:1 ratio, and 15% paraformaldehyde was added to a final concentration of 4% paraformaldehyde. The cells were fixed at 4°C overnight. On the next day, cells were centrifuged at 200×*g* for 5 min to remove paraformaldehyde and then washed with PBS and centrifuged at 50×*g* for 5 min. The washing was repeated for 5 to 7 times until individual red blood cells were not the main population checked under the microscopic. Visible red clots were removed during washing. After a final centrifugation and removal of the supernatant, the cellular fraction was fixed again with 4% paraformaldehyde at a ratio of 1:4 (original blood volume:fixative volume) and dropped (150 μm/drop from ~8 cm above the slide) onto gelatine-coated histology slides.

### Cellular clump isolation

Blood was collected from 8- to 10-week-old Balb/C, 8- to 10-week-old c57BL/6 or 8- to 16-week-old GFP transgenic mice via cardiac puncture after CO_2_ euthanasia. Erythrocyte lysis buffer (155 mM ammonium chloride, 10 mM potassium hydrogen carbonate, and 0.1 mM EDTA) was added to mouse blood. The lysates were incubated for 20 min with gentle mixing. Naturally precipitated spheroids were collected from the bottom of the tube after removing the upper lysate. The precipitant was then washed 3 times with PBS in a narrow-bottom tube and collected via natural precipitation for 1 min each time intervals. Any visible red-coloured blood clots were removed during the washes. Light-pink–colored cellular clumps were cultured or fixed for histology.

### Bone-marrow cell isolation

Balb/C or c57BL6 mice were euthanized with CO_2_. Femurs and tibias were collected. After removing the attached tissues and a rinsing in PBS, the femurs and tibias were cut open at the both ends. The bone marrow was flushed by using PBS into collecting tubes with centrifugation. The bone-marrow cells were immediately fixed for histology.

### Ischemic kidney damage

In total, 54 Balb/C mice were used for experiments. Eight- to 10-week-old female Balb/C mice were anaesthetized with pentobarbital (70 mg/ kg body weight) via intraperitoneal injection. The level of anesthesia was assessed by: respiration rate, positive toe pinch, and corneal reflex. All mice were placed on a heating pad during the surgery and the recovery time to maintain the body temperature. After hair was shaved, the abdominal skin was cleaned and opened under sterilized condition. Kidney arteries on each side were ligated at the same time with use of a 4–0 suture. After 45 min, sutures were removed to reopen the ligated arteries before the abdomen skin was closed. Mice were monitored every 15 min for 2 hours. Post-operative analgesia was accomplished with buprenorphine 0.05 mg/kg to stop suffering and distress. Mice in obvious distress, such as weight loss more than 15%, mobility loss, failure to groom, failure to show normal inquisitiveness were euthanized by CO^2^. Mice were euthanized at day 1 and weeks 1, 2, 3, 4, and 6 after transplantation. Kidneys were removed and fixed for histology. At least 3 mice were used for each time point.

### TUNEL assay

The ischemia-damaged kidneys were fixed, embedded and sectioned. Briefly, after de-waxing, rehydration and washing, sections were treated with proteinase K (25 μg/ml in tris-CaCl2 buffer) at 37°C for 12 min. Then, slides were placed in 0.1 M glycine for 5 min to stop the reaction. After washing, slides were reacted with TUNEL assay solution containing terminal deoxynucleotidyl transferase (TdT) and nucleotide mixture for 60 min. Positive control slides were treated with DNAase (30U/ml PBS) for 10 min at room temperature before reacting with TUNEL assay solution. Negative control slides were treated with nucleotide mixture only. After washing, the slides were examined under a fluorescence microscope.

### Haematoxylin and eosin (H&E), DAB, and fluorescence staining

Cellular portions dropped on cover slides were fixed for H&E staining. The natural precipitated cellular clumps were embedded and sectioned for H&E or immunofluorescence studies. For immunofluorescence staining, sectioned clumps were blocked, then incubated with different antibodies at dilutions ranging from 1:50 to 1:100 in blocking buffer overnight at 4°C, then washed and incubated with a fluorescent conjugated secondary antibody for 1 hr. After a washing, cells were counterstained with DAPI and photographed by conventional fluorescence microscopy. Control tissue was treated with nonspecific antiserum from the same species of primary antibody. Staining was visualized by standard fluorescence microscopy (Leica).

### Comparison of cells by age of mice

Blood was collected from 4 of 8-week-old and 3 of 40-week-old Balb/C mice. An amount of 0.5 ml of blood from each sample was transferred into a 15 ml centrifuge tube and immediately diluted with 1x PBS at a ratio of 1:5 (blood:PBS), then centrifuged at 200×*g* for 5 min. After removing the supernatant, the cellular portion was diluted with PBS at a 1:1 ratio, and 15% paraformaldehyde was added to a final concentration of 4% paraformaldehyde. The cellular portion was fixed at 4°C overnight and next day were washed at 50 xg for 5 times. After a final centrifugation and removal of the supernatant, the cellular fraction was fixed again with 4% paraformaldehyde at a ratio of 1:3 (original blood volume:fixative volume). The prepared cellular portions (~2 ml/per sample) were dropped onto gelatine-coated histology slides. Each slide was checked under a microscope by using a line-scanning method. Particle-producing cells with specific morphology were counted. The differences in cells between the two age groups were analyzed by 2-tailed Student *t* test with Microsoft Excel 2008.

## Results

### Identifying coiling-tube-like cells that contain numerous small particles

**Fig 1:**
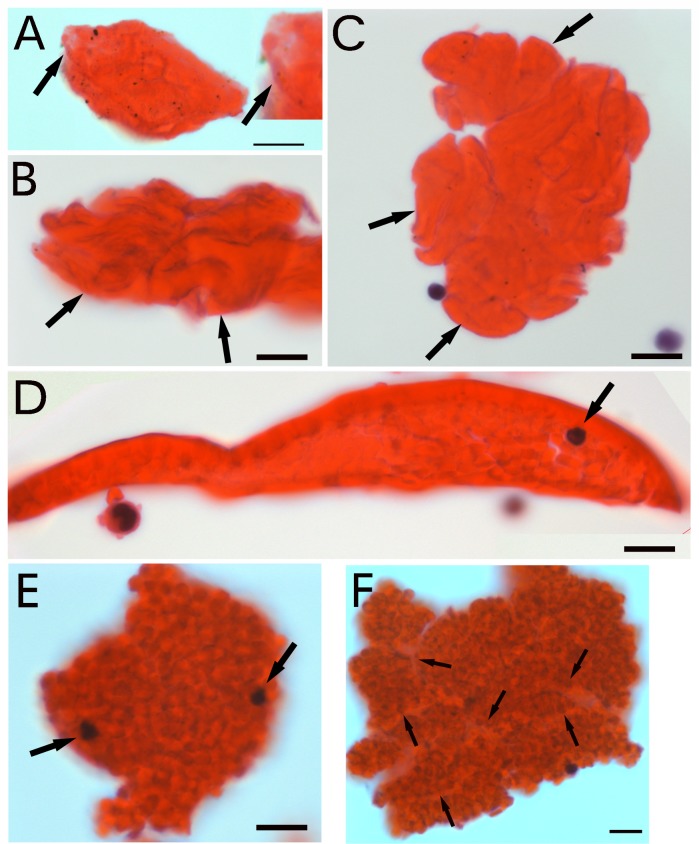
Identifying large cells that produce numerous small particles. Cellular portions from blood of GFP-transgenic mice (A) or bone marrow of C57BL/6 mice (B-C) were dropped on slides and stained with hematoxylin and eosin (H&E). A thin transparent membrane was observed on the surface (arrows in A and the amplified image). The larger cellular structures might be from multiple smaller-one connections (arrows in B, C). Cellular portions from GFP transgenic mice were embedded, sectioned, and stained with H&E to observe large cells (D). This cell had a tightly particle-connected outer layer and contained numerous small red particles. Small “naked” nuclei were identified (arrow in D). Cellular portions of 8- to 10-week old Balb/C mice were dropped onto slides. (E) A large particle-producing cell was identified. Two small “naked nuclei were observed inside the cell (arrows). (F) A cellular cluster composed of tightly attached particle-producing cells. The thin lines (arrows) show the membrane attachment. Bars = 10 μm.

Cellular portions isolated in blood or bone marrow were dropped on slides, stained with H&E and examined by microscopy. Large cellular structures in blood (Fig 1A) and bone marrow (Fig 1B and 1C), from 30 to 200 μm were identified. The larger sized might from the adhesion of multiple smaller ones (each was pointed by arrows in Fig 1B and 1C). They stained strongly with eosin, which suggested that they were rich in proteins. A thin transparent membrane was identified on the surface of the smaller cellular structure (arrows in Fig 1A). Except for the thin membrane, the surfaces of these cells were not smooth, with the structures similar to the coiled thin tubules; however, because of the dense red color, the detailed structures of these cells were difficult to identify. To observe the detailed structures, cellular samples were embedded, sectioned and examined. Microscopy revealed a section of a large cell that contained numerous small red particles (Fig 1D) and had a tight particle-connected outer layer, with a few “naked” small nuclei inside (arrow in Fig 1D) that did not stain for CD4, the lymphocyte marker (data not shown). Not all individual particles could be clearly identified inside these cellular structures. Without sectioning, we also identified large cellular structures that contained numerous small particles (Fig 1E and 1F), which suggested that the outer surfaces of these larger cells were lost. These cells attached to each other to form a cellular clump. Thin lines were identified on the cellular clump (arrows in Fig 1F), which indicated multiple cell attachment. Our data suggest that the appearance of these particles in the large cells depended on the differentiation degree of these cells.

In addition, the number of particle-producing cells was greater in 8- than 40-week-old mice but not significantly, possibly because of individual differences and few mouse numbers (S1 Fig).

### Small red particles aggregate and fuse into cells

**Fig 2:**
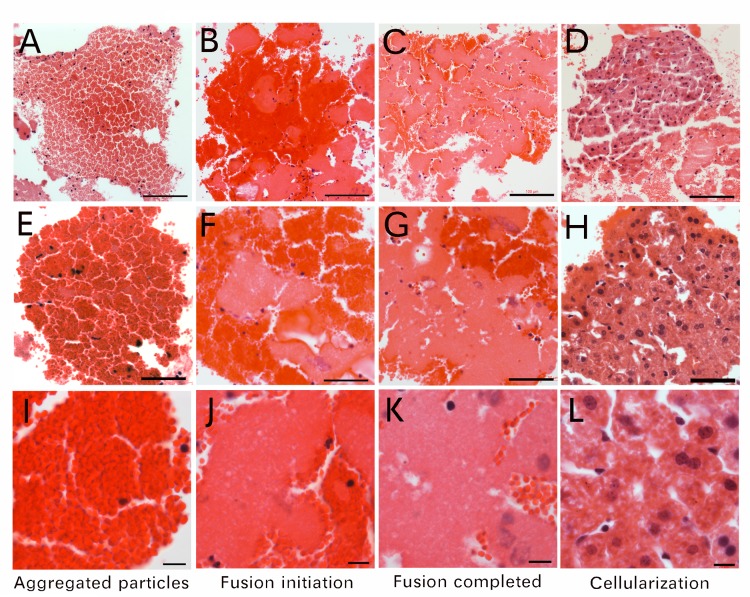
Small particles aggregate, then fuse into cells. Isolated particle-composed clumps were examined under 20x (A-D), 40x (E-H) and 100x (I-L) lenses. The column on the left (A, E, I) shows only aggregated particles. The second column (B, F, J) shows particle fusion initiation. The third column (C, G, K) shows fusion completion. The column on the right shows cellularization (D, H, L). The high-magnified image revealed small particles arranged in groups; the size of each group was similar to a cell size (I). Bar in A-D = 100 μm; E-H = 50 μm; I-L = 10 μm.

Large cellular clumps were sectioned, stained and examined. We found that some large cellular clumps were composed of not cells but numerous small particle aggregations (Fig 2A, 2E and 2I). These particle-composed clumps could reach close to 400 μm in diameter (Fig 2A). These small particles in one clump were the same size and were arranged in groups, the size of each group similar to that of a cell (Fig 2I). In other clumps, particle fusion was initiated in the center of the grouped particle clumps (Fig 2B, 2F and 2J). Also, we have observed that in some clumps, grouped particle fusion was completed (Fig 2C, 2G and 2K), excepting traces small particles was still visible. Furthermore, these fusion-derived cells completed cellularization and become the regular nucleated cells (Fig 2E, 2H and 2L). Under lower magnification (Fig 2A–2D), the particles or the particle-derived cells in each clump were in the same differentiation status, which suggests that the cellularization procedure of these particles were in a synchronized pattern in each clump. Some small particles could still be seen at the peripheral areas when the particle clumps become cellular clumps (Fig 2D and 2H), which indicate that they originated from small particles. We believe that multiple particle-producing cells with similar maturation were connected, fused and differentiated into large nucleated cellular clumps.

### Particle-fusion–derived cells are epithelial cells

**Fig 3:**
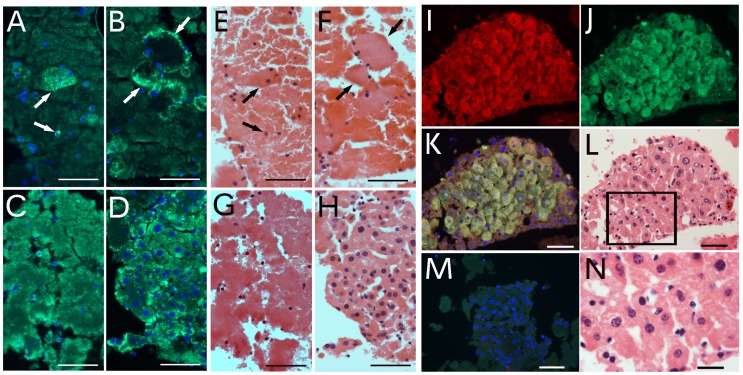
The particle-fusion–derived cells are epithelial cells. E-cadherin expression was examined in the particle-aggregated clump (A), fusion initiation stage (B), complete fusion stage (C) and cellularized clumps (D). The same slides were again stained to H&E after the fluorescent images were taken. The H&E images in E-H are the same areas of A-D, respectively. The arrows in A point to the same areas as in E, and in B to the same areas as in F. A cellular clump was further stained for E-cadherin (I, red) and GFP (J, green) and merged images (K). The same sections were stained with H&E after fluorescence images were taken (L). The image in N was the amplification of the square in L. The control image was from the non-specific anti-sera (M). Bars in A-M = 50 μm, in N = 20 μm.

We examined the expression of epithelial-specific marker E-cadherin on the particle- or cellular-composed clumps from particle aggregation to cellularization. In one particle-composed aggregate, E-cadherin was expressed on a few small cells and one group of aggregated particles (arrows in Fig 3A). In the fusion initiation period, E-cadherin was expressed mainly at the edges of the fused particles (arrows in Fig 3B). E-cadherin was expressed on most fused areas when the fusion was completed (Fig 3C) and expressed on most cells during cellularization (Fig 3D). The same slides were stained to H&E after the fluorescent images were taken (Fig 3E–3H). The results further supported that E-cadherin was expressed as early as the fusion started in the aggregated particles. A newly formed cellular clump was further examined (Fig 3I–3L). Except for peripheral areas, most cells in the particle-fusion–derived cellular clump were positive for E-cadherin and GFP. The same sections were stained with H&E after fluorescent images were taken (Fig 3L) and its amplified image (Fig 3N). The results confirmed that this cellular clump was composed of only epithelial cells that originated from the particle-producing cell or cells. The data also indicate that particle-derived epithelial cells can be formed in the blood and delivered to the target tissue via blood circulation.

### Kidney-duct epithelial-cell regeneration involved the particle-fusion mechanism

**Fig 4:**
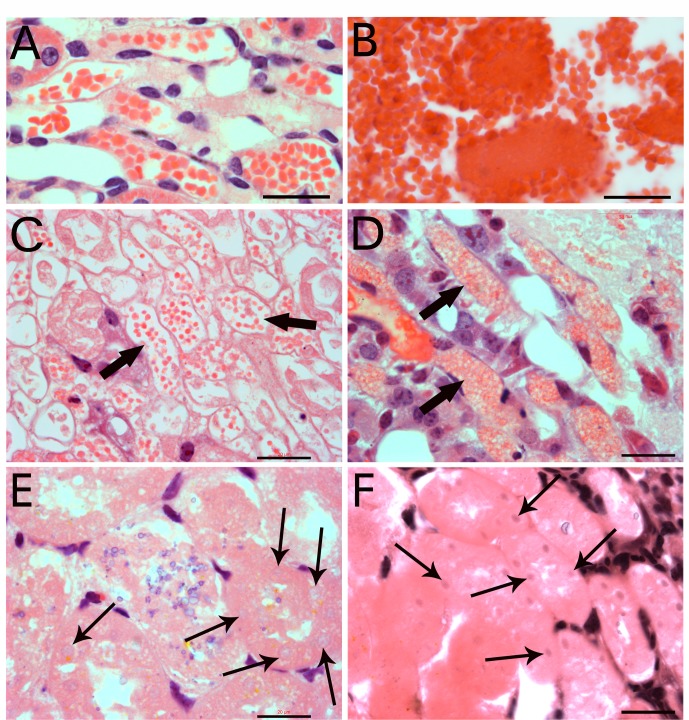
Small particles appeared in ischemic kidney ducts. Balb/C mouse kidney-duct epithelial cells were regenerated via the particle-fusion mechanism after ischemia damage. Microscopy revealed that the particles in kidney regeneration (A) had the similar sizes to those from the blood (B). Numerous small particles appeared in mouse kidney ducts 1 day after kidney ischemia damage (arrows in C). Particle fusion was observed in the regenerating kidney ducts 1 week later (arrows in D). Small nuclear fragments appeared in the regenerating kidney ducts (thick arrows in E); and diluted nuclear materials with sizes similar to a nucleus appeared (thin arrows in E). Also, new nuclei characterized as small, faint, and well arranged along the duct appeared in the regenerating ducts (thin arrows in F). Bars = 20 μm.

To identify whether the phenomenon of particle-fusion to produce epithelial cells occurs in *in vivo*, we examined the regeneration of mouse kidney ducts after ischemia damage by surgical ligation of kidney arteries and reperfusion. More than 50% of kidney tissue showed ischemia damage (S2 Fig). In a 1-week damaged kidney, we observed similar particle fusion in regenerating kidney ducts that followed by nuclear programming in *in vivo* mouse studies. Under the same magnification, microscopy revealed that small particles appearing in kidney-duct regeneration (Fig 4A) were similar in size to the particles identified from blood (Fig 4B). These particles appeared in mouse kidney ducts 1 day after kidney ischemia damage (Fig 4C). The small particles in a 1-day ischemia-damaged kidney were morphologically different from erythrocytes in an undamaged kidney (S3 Fig). Although both stained red for eosin, erythrocytes were disk-like and depressed in the center or were flat side next to flat side to form a stack. The small particles were round in all directions and did not show the center depression, or had solid contents. By 1 week after damage, regenerating kidney ducts showed particle fusion (Fig 4D). In addition, nuclei with weak staining for haematoxylin but with similar sizes as the regular nucleus appeared in the particle-fused materials (Fig 4E). Furthermore, new nuclei characterized as small, faint, and well arranged along the duct appeared in the regenerating ducts (Fig 4F). All these regenerative processes could be observed in 1-week ischemia-damaged kidney ducts (S4 Fig). TUNEL assay of damage kidneys (S5 Fig) revealed that nuclei with weak staining for haematoxylin (Fig 4E) were apoptotic nuclei (arrows in A and B in S5 Fig). However, because of the small sizes of the DNA fragments, it was difficult to trace the apoptosis. Thus, kidney-duct epithelial cells could be regenerated via the particle-fusion mechanism. These small particles migrated via the blood circulation to the damaged kidney ducts. The nucleus is formed inside the fused small particles by a still unknown mechanism.

### Naked nuclei and DNA fragments participate in nuclear programming

**Fig 5:**
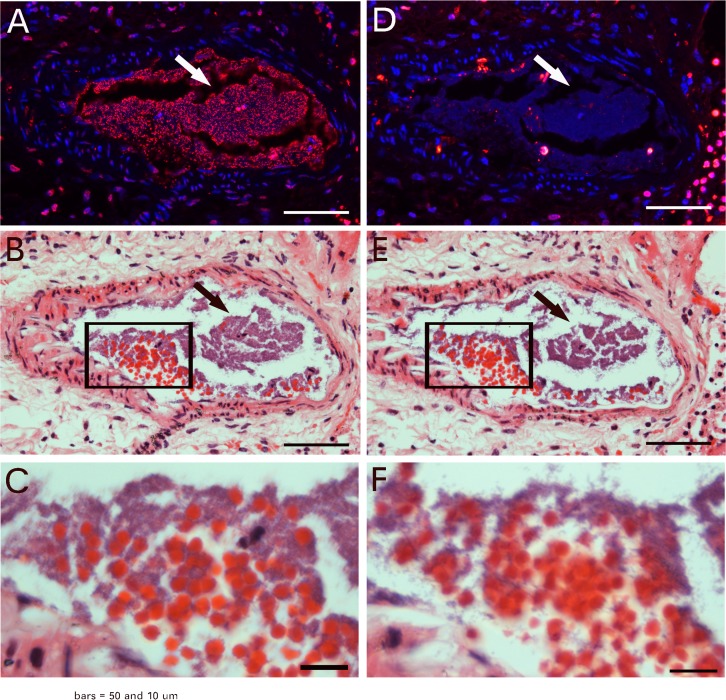
Sand-like DNA materials in circulation. Kidneys were collected 1 day after ischemia damage. Positive control slides were pretreated with DNAase before TUNEL assay. An area (arrow in A) with strong positive staining was observed on the positive control slide (A). The same area (arrow in D) did not show stains on the regular slide (D). H&E staining revealed that this strong-stained area was in a blood vessel (B, E). High-magnified microscopy (rectangle areas in B, E) revealed that these DNA materials in the blood vessel were tiny sand-like or thin fiber-like (C, F). Bars in A-B, D-E = 50 μm, in C, F = 10 μm.

**Fig 6:**
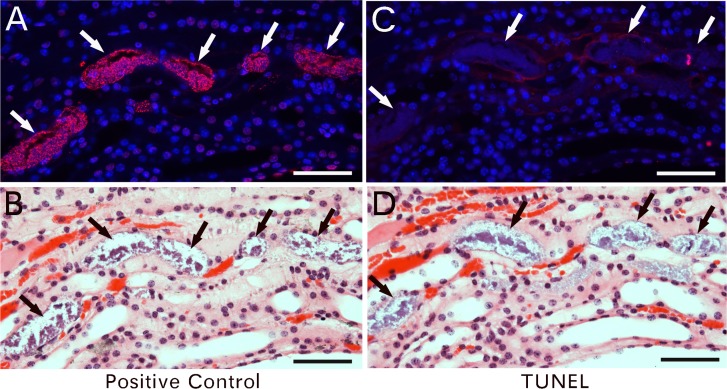
Sand-like DNA particles migrate into kidneys for regeneration. Besides blood vessels, small sand-like DNA materials were identified in tissues of renal papillae. A slide pre-treated with DNAase showed grouped sand-like DNA particles that were apoptotic (arrows in A and B). Slides not pre-treated with DNAase showed no apoptosis (arrows in C and D). Bars = 50 μm.

Although how the nucleus is formed or programmed in the newly made cells is still unclear, we found small DNA particles delivered via circulation to damaged kidneys (Fig 5). The 1-day ischemia-damaged kidney section pretreated with DNAase before TUNEL assay showed strong positive staining (Fig 5A) in materials located inside a blood vessel (Fig 5B and 5C) in the kidney pelvis area (arrow in A in S6 Fig). In comparison, these DNA materials in the same area did not show positive TUNEL staining (Fig 5D–5F). High-magnified microscopy revealed that these DNA materials in blood vessels were tiny sand-like or thin fiber-like, possibly the “Dot Cells” identified by the author in previous report [19]. Most of these sand-like DNA particles were aggregated and closely adjacent to the small epithelial particles (Fig 5C and 5F). Similar DNA materials were observed in kidney duct areas of the renal papillae (arrow in B in S6 Fig). With DNAase pre-treatment, these sand-like DNA materials grouped together and migrated along the damaged tissues (arrows in Fig 6A and 6B). Without DNAase pre-treatment, staining was negative (arrows in Fig 6C and 6D). Because these materials were delivered to the kidney after the ischemia damage, they did not show any apoptosis. Positive control sections were pretreated with DNAase and therefore showed strong DNA damage. Therefore, after the ischemia damage, the materials delivered via the blood circulation included small particles for kidney duct regeneration and also DNA materials for nuclear remodeling of new cells. We believe that these circulating DNA fragments participate in nuclear remodeling. However, because of their small size and the limited techniques, we still cannot purify these small-sized DNA particles.

### The newly formed epithelial cells are stem cells

**Fig 7:**
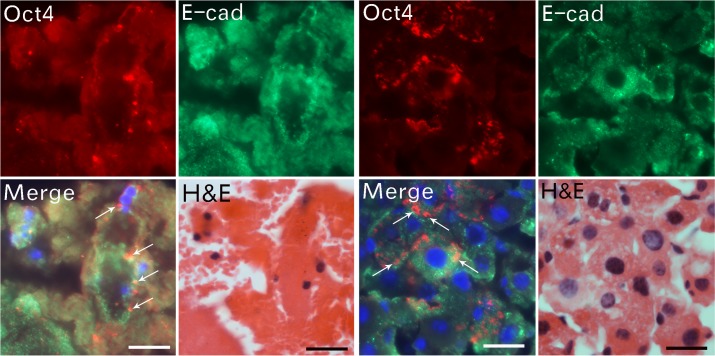
Externally derived Oct4 participates in epithelial cell cellularization. The expression of Oct4 (red) and E-cadherin (green) was examined in the partly particle-fused clumps (left 4 panels). The merged image shows Oct4 expressed on the outer edge of the fused particles (thin arrows). H&E staining of the same slide in fluorescent images show Oct4 (red) and E-cadherin (green) colocalization in the cellularized clumps (right 4 panels). The merged image shows Oct4 expressed on the alveoli-like structures in the cytoplasmic areas of the newly formed epithelial cells (thin arrows). The same slides were stained with H&E after fluorescent images were taken. Bars = 20 μm.

In order to see if the newly formed epithelial cells were stem cells, we examined the co-localization of Oct4 (Fig 7A and 7E) and E-cadherin (Fig 7B and 7F) expression from particle aggregation to cellularization. The same slides were stained to H&E after the fluorescent images were taken (Fig 7D and 7H). We found that Oct4 was expressed as early as the particle fusion initiated. It expressed at the edges of the fused particles and some peripheral areas of the clumps (thin arrows in Fig 7C). Oct4 expressed as small particles, which suggest that Oct4 maybe carried by very small unknown carries to the aggregated particles. However, the carriers may be too small to be identified. During the cellularization, Oct4 was expressed on the alveoli-like structures in the cytoplasmic areas (thin arrows in Fig 7G), instead of in the nucleus of the newly formed cells. These data again supported that Oct4 was derived externally by some unknown carries that exist naturally in the blood.

### DNAs located in the cytoplasmic area in newly formed cells

**Fig 8:**
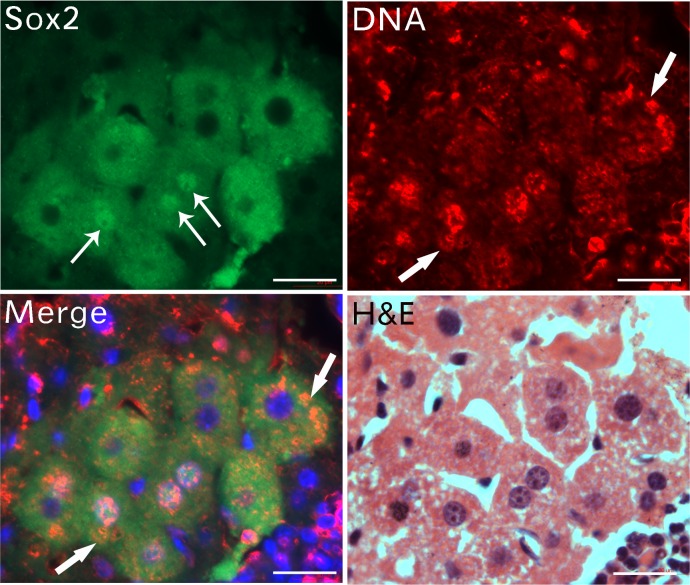
The cytoplasmic areas of the newly formed cells contain DNAs. Sox2 expressed in both the cytoplasmic and nuclear areas (arrows) of a group of cells. Besides other cells, DNA was expressed in Sox2-expressing cells in alveoli-like patterns in cytoplasmic areas (arrows). H&E staining showed these Sox2-expressing cells newly made from the small particle fusion. Bars = 20 μm.

It is well known that both Oct4 and Sox2 are transcription factors and locate in the nucleus. However, we have repeatedly found both Oct4 and Sox2 in cytoplasmic areas of the newly formed cells, besides the nucleus. To further investigate, we examined DNA expression in newly formed cells (Fig 8). In a cellular clump, Sox 2 was expressed in both cytoplasmic and nuclear areas in a group of cells. DNA was also expressed in similar areas of these cells, except the peripheral cells. In some cells, DNA was expressed in alveoli-like patterns in cytoplasmic areas. Hence, the newly formed cells contained DNA in cytoplasmic areas. As well, the aggregated particles contained both small particles and DNA materials that may be from “naked” nuclei or from sand-like DNA materials in circulation. These data provide evidence that cells can be formed by particle aggregation.

### Particle-fusion–derived cells become epithelial-specific stem cells

**Fig 9:**
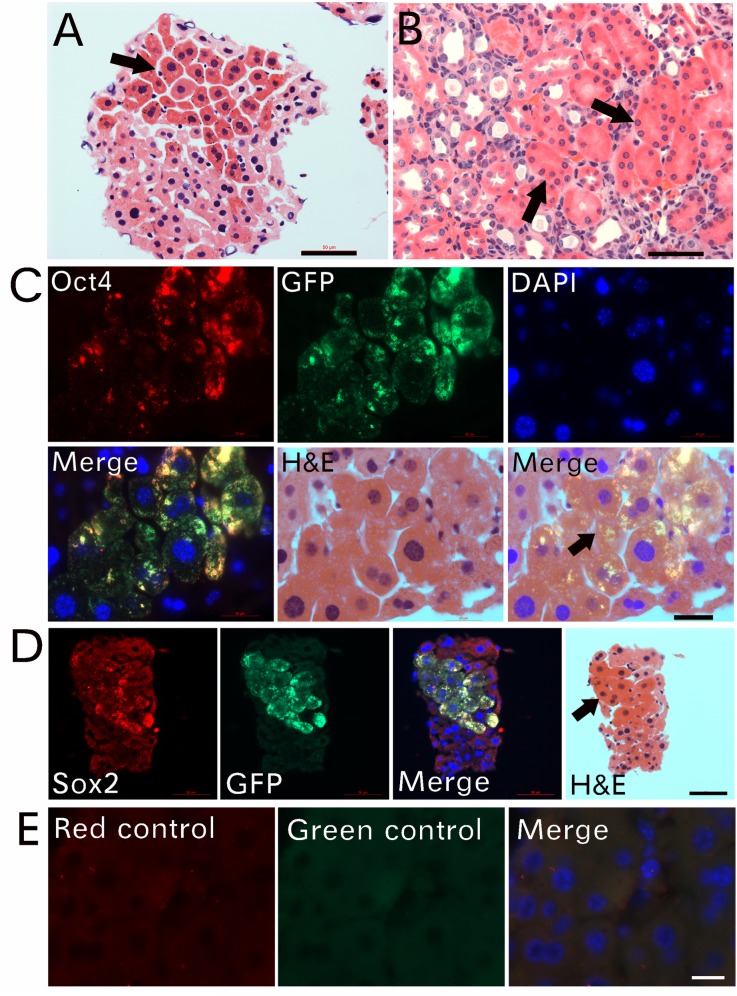
Particle-fusion–derived cells become epithelial-specific stem cells. Cellular clumps isolated from GFP-transgenic mice were used. The cells in the center of particle-fusion–derived cellular clumps stained rich with eosin (arrow in A). Rich eosin-stained epithelial cells appeared in newly regenerated kidney ducts (arrow in B). Higher magnification revealed that Oct4 and GFP (C) or Sox2 and GFP (D) were co-expressed only in cells with rich eosin staining (arrows in C, D). Cells with less eosin staining located mainly at the periphery and did not express Oct4 or Sox2 and GFP. Control sections are in E. Bars in A-B, D = 50 μm; in C, E = 20 μm.

We observed that cells in the center of some particle-fusion–derived cellular clumps stained richer with eosin (Fig 9A), which suggests that these cells contained more proteins than did cells at the peripheral areas. Similar rich eosin-stained epithelial cells were identified in newly regenerated kidney ducts (Fig 9B). These epithelial cells located in kidney ducts, the lumens of which had not yet opened, which suggests that these kidney ducts may not function or are not fully differentiated. To identify the differences between the more- and less-eosin–stained cells, we examined the expression of these stem cell markers in cellular clumps isolated from the blood of GFP-transgenic mice. Under lower magnification (S7 Fig), GFP was expressed strongly in eosin-rich–stained cells. Similarly, Oct4, except for a few cells, was expressed in these cells. Higher magnification revealed that Oct4 and GFP (Fig 9C) or Sox2 and GFP (Fig 9D) were co-expressed only in cells with more cytoplasmic proteins, with more-intense eosin staining. Cells with less-intense eosin staining, located mainly at the periphery, did not show strong expression of Oct4 or Sox2 and GFP. These observations suggest that although particle-fusion–derived new cells contain DNA, they need to reach a certain degree of maturity before they can express Oct4, Sox2 and GFP.

## Discussion

Our data clearly show that a group of cellular structures produce and release small particles in blood circulation. These particles migrate to the damaged kidneys, or other damaged tissues to regenerate the epithelial cells. The aggregated particles showed synchronized differentiation, appearing as particle fusion followed by nuclear programming, thereby producing new E-cadherin–expressing epithelial progenitor cells. This epithelial cell-forming procedure occurs in the circulation and specific tissues. By passing through the above stages, one particle-composed clump can eventually differentiate into a large cellular clump composed of numerous epithelial cells for tissue need. Newly formed cells further differentiated into Oct4- and Sox2-expressing stem cells that may have high proliferation activities. By this procedure, small particles consistently make new epithelial progenitor cells to provide the sufficient stem cell needs of the body. Our results provide evidence that specific epithelial progenitors can be made from particle-fusion–derived cellularization. Thus, the epithelial progenitor/stem cells originate from these large cellular structures.

Data from *in vivo* mouse kidney duct regeneration revealed that epithelia of kidney ducts were regenerated by the same mechanism of particle-fusion followed by nuclear programming. Fast-renewing epithelial cells such as in skin, intestine, kidneys and liver may all derive from circulating small particles. However, even though our data provide evidence that these large cellular structures are upstream of the epithelial cells and produce similar-sized particles to those found in kidney, our data could not provide evidence of that these large cellular structures are identical and the particles they produced are the origin of all types of epithelia cells. In addition, except for kidney, we do not know which epithelia region or tissue is the destination of these blood particle-fusion–derived epithelial cells. Further investigations will be performed to elucidate these questions.

We believe that at least three different sources of DNA materials are involved in the nuclear remodeling of newly formed cells. First, nuclear remodeling may be regulated by the small “naked” nuclei that locate together with these particles in the large cells. We observed a few small “naked” nuclei inside the particle-producing large cellular structures together with small particles (Fig 1E, Fig 2I). These nuclei were not classical cell nuclei because they were similar in size to the small red particles and contained almost no cytoplasmic materials. The roles of these “naked” nuclei in nuclear remodeling may be fusion with many small particles or releasing DNA fragments that provide the genetic information to the fused particles for regeneration into specific cell lineages. These hypotheses are supported by data in Fig 3A showing an E-cadherin–expressing small nucleus. The image in Fig 3E clear shows that this small nucleus was embedded in the small particles, which indicates that this nucleus was not exogenous. The fused particles possibly contain regulatory factors for DNA polymerization, transcription and nuclear formation. Second, we do not exclude that the small apoptotic DNA fragments in damaged kidneys were recycled for the nuclear remolding. This hypothesis is supported by the observation of others describing nuclear programming induced by trace amounts of DNA more than 20 years ago: a 5-ng small amount of phage DNA could form a new nucleus after injection into Xenopus eggs [20]. The assembly of the mini nuclei does not depend on a specific DNA sequence and does not require the presence of the egg nucleus, centromere or telomere [20]. Third, numerous sand-like DNA materials (Fig 5, S6 Fig, S7 Fig) in circulation may participate in nuclear remodeling because the time of their appearance paralleled that of the small particles. The small-sized DNA materials may contain “Dot Cells” that expressed E-cadherin and could reduce scarring in a previous report [19]. However, the relation of the “Dot Cells” and the small red particles in epithelial regeneration needs to be further investigated. Therefore, *de novo* nuclear programming can occur with a small amount of DNA, and egg materials are essential for nucleus formation. We believe that the particle fusion-derived materials contain the egg materials. Thus, the DNA fragments in particle-fusion–derived material may be via a similar mechanism for nuclear remodeling.

In another way, our data support that epidermal stem cells do not self-renew via asymmetric division [18]. Our data demonstrate that epithelial stem cells are newly made from multiple small particles in the blood or bone marrow. These small particles are constantly carried to specific tissues under the physiological condition or during an emergency. We also identified these particle-producing cells in human blood (S8 Fig). The data support our hypothesis that epithelial stem cells are produced with a similar mechanism in humans. From our preliminary data, we do not know whether human blood has as many small-particle–producing cells as mouse blood, because mice have stronger regenerative activities than humans.

Our data also provide important information for epithelial tissue regeneration and the potential clinical application. Transplantation of small particles may have better clinical potential in epithelial cell regeneration than using the mature epithelial stem cells. These small particles are not cells, hence, do not have much genetic information, and have few membrane-recognized markers. In addition, their small size can also avoid cell clots caused by cell membrane adhesion during cell transplantation. The characterizations of the small particles provide much safer standards for their uses in the clinical than using the regular cells or stem cells. Finally, our data indicate that blood components are far more complicated than what we previously knew.

## Supporting information

S1 Fig More particle-producing cells in the blood of young than old mice. Particle-producing cells in 8- and 40-week-old mice analyzed by Student *t* test.
(TIF)

S2 Fig Evaluation of ischemia damage in normal kidney and after 1-day ischemia damage. (TIF)

S3 Fig The morphology of erythrocytes and red small particles in normal kidney and 1-day ischemia-damaged kidney. Bars = 20 μm.
(TIF)

S4 Fig Particle aggregation and fusion in 1-week ischemia-damaged kidney (A). Amplified images show particle aggregation near the pelvis area (B, arrows in C). Particle fusion in the kidney duct-like structures near the cortex (D, arrows in E). Bars in B, D = 100 μm; in C, E = 20 μm.
(TIF)

S5 Fig Weak-stained nuclei were apoptotic. Sections of 1-week ischemia-damaged kidney were stained by TUNEL assay. Nuclei that stained strongly red were apoptotic cells (A). H&E staining of the same section revealed that weak haematoxylin-stained nuclei were apoptotic cells (arrows in A and B). Bars = 50 μm.
(TIF)

S6 Fig Sand-like DNA materials appeared in kidney pelvis after 1-day ischemia. Low magnified images show 2 samples of 1-day ischemia-damaged kidneys. The arrow in A is the blood vessel described in Fig 5. The arrow in B is the grouped sand-like DNA materials described in Fig 6. Bars = 1mm.
(TIF)

S7 Fig A whole cellular clump was imaged to compare the differences in octamer-binding transcription factor 4 (Oct4) and GFP expression in eosin-rich and less-stained cells. GFP was expressed only in eosin-rich–stained cells. Except for a few cells, OCT4 was also expressed in these cells. Bar = 50 μm.
(TIF)

S8 Fig Human blood was collected from a volunteer at age 28. The cellular portion was dropped on slides and stained with H&E. Particle-producing cells were identified and imaged. Bar = 20 μm.
(TIF)
