# Supplementary figures and images for: Epithelial stem cells are formed by small-particles released from particle-producing cells
Source: PLoS One. 2017 Mar 2;12(3):e0173072. doi: 10.1371/journal.pone.0173072 (PMC5333853; doi:10.1371/journal.pone.0173072)

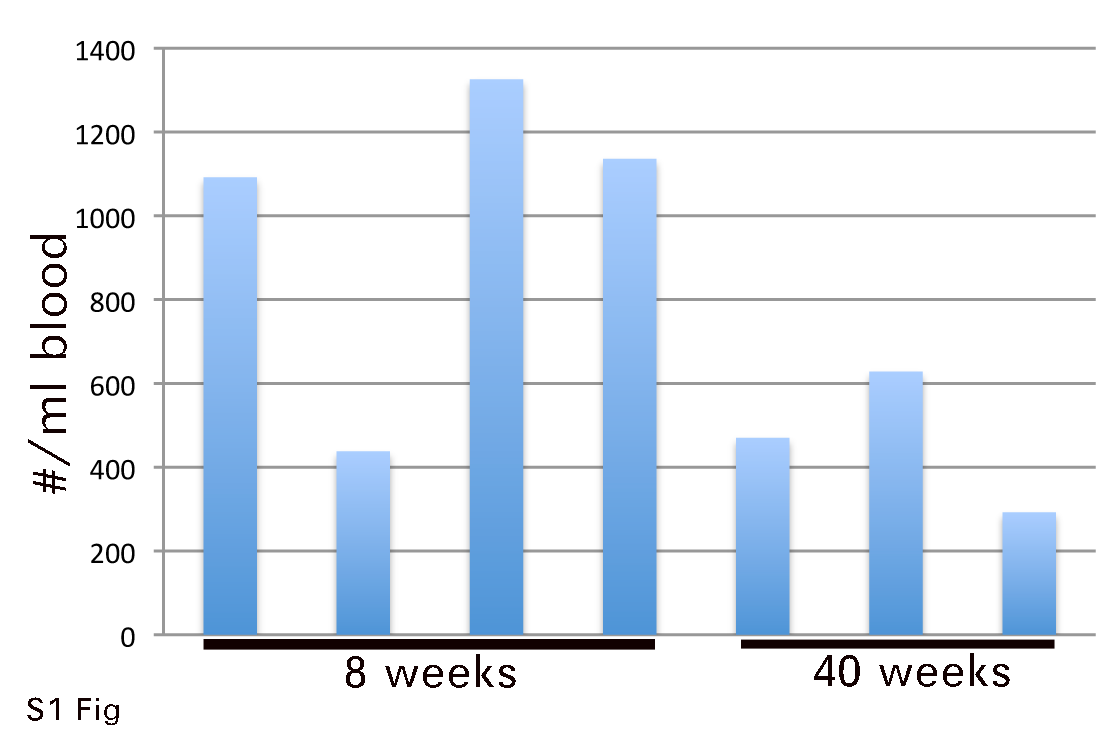

Supplement: S1 Fig — Particle-producing cells in 8- and 40-week-old mice analyzed by Student t test. (TIF) [file pone.0173072.s001.tif]

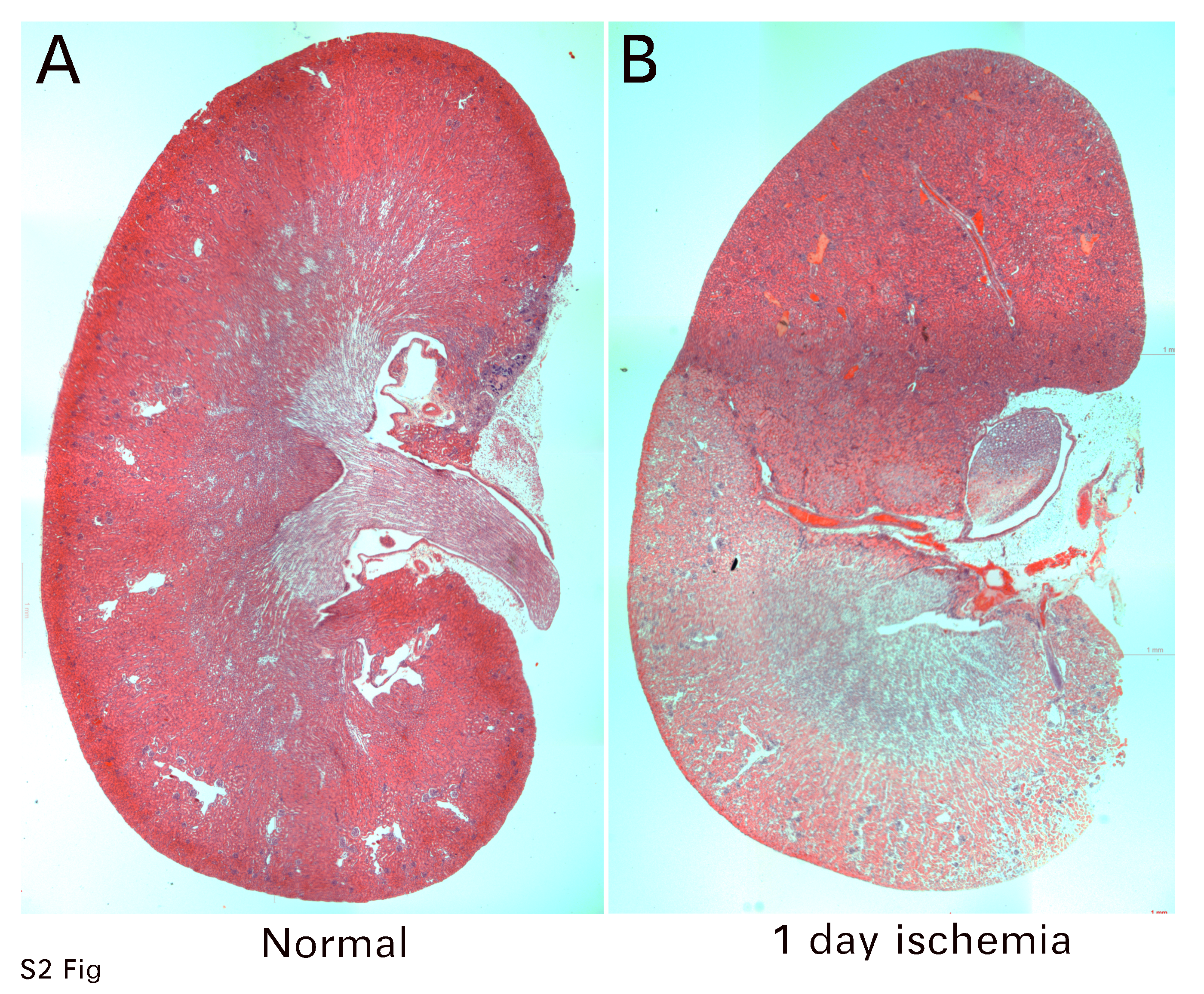

Supplement: S2 Fig — (TIF) [file pone.0173072.s002.tif]

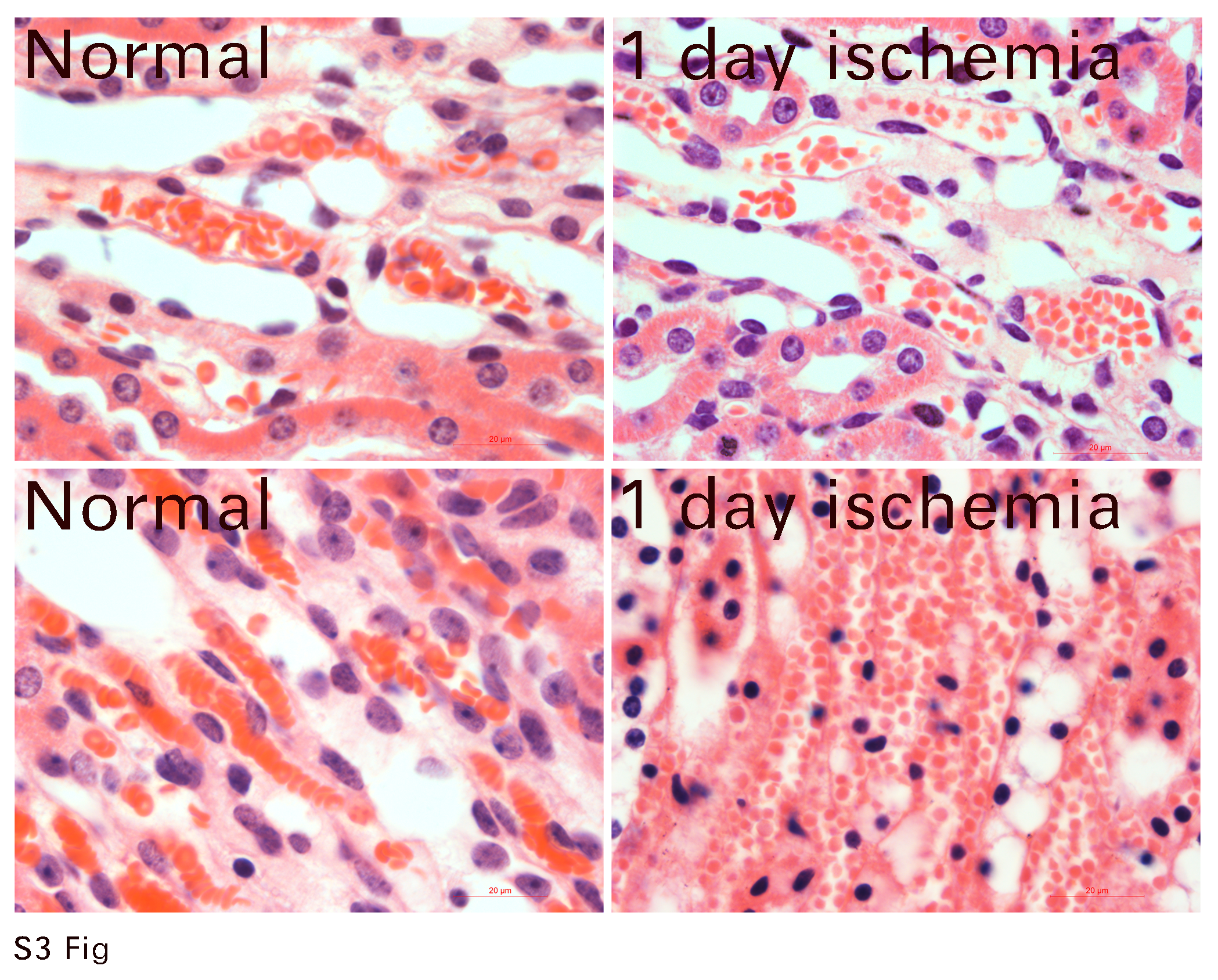

Supplement: S3 Fig — Bars = 20 μm. (TIF) [file pone.0173072.s003.tif]

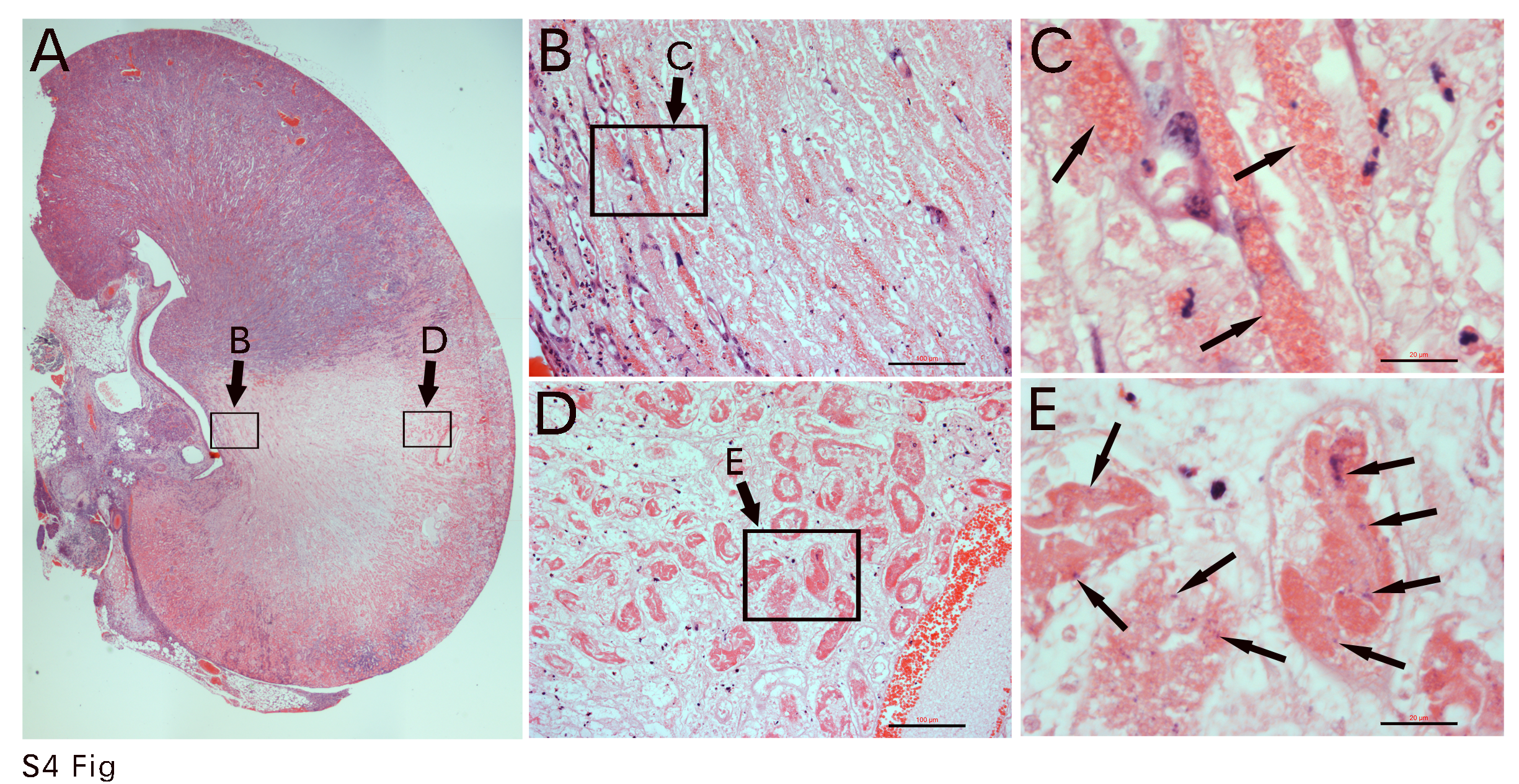

Supplement: S4 Fig — Particle aggregation and fusion in 1-week ischemia-damaged kidney (A). Amplified images show particle aggregation near the pelvis area (B, arrows in C). Particle fusion in the kidney duct-like structures near the cortex (D, arrows in E). Bars in B, D = 100 μm; in C, E = 20 μm. (TIF) [file pone.0173072.s004.tif]

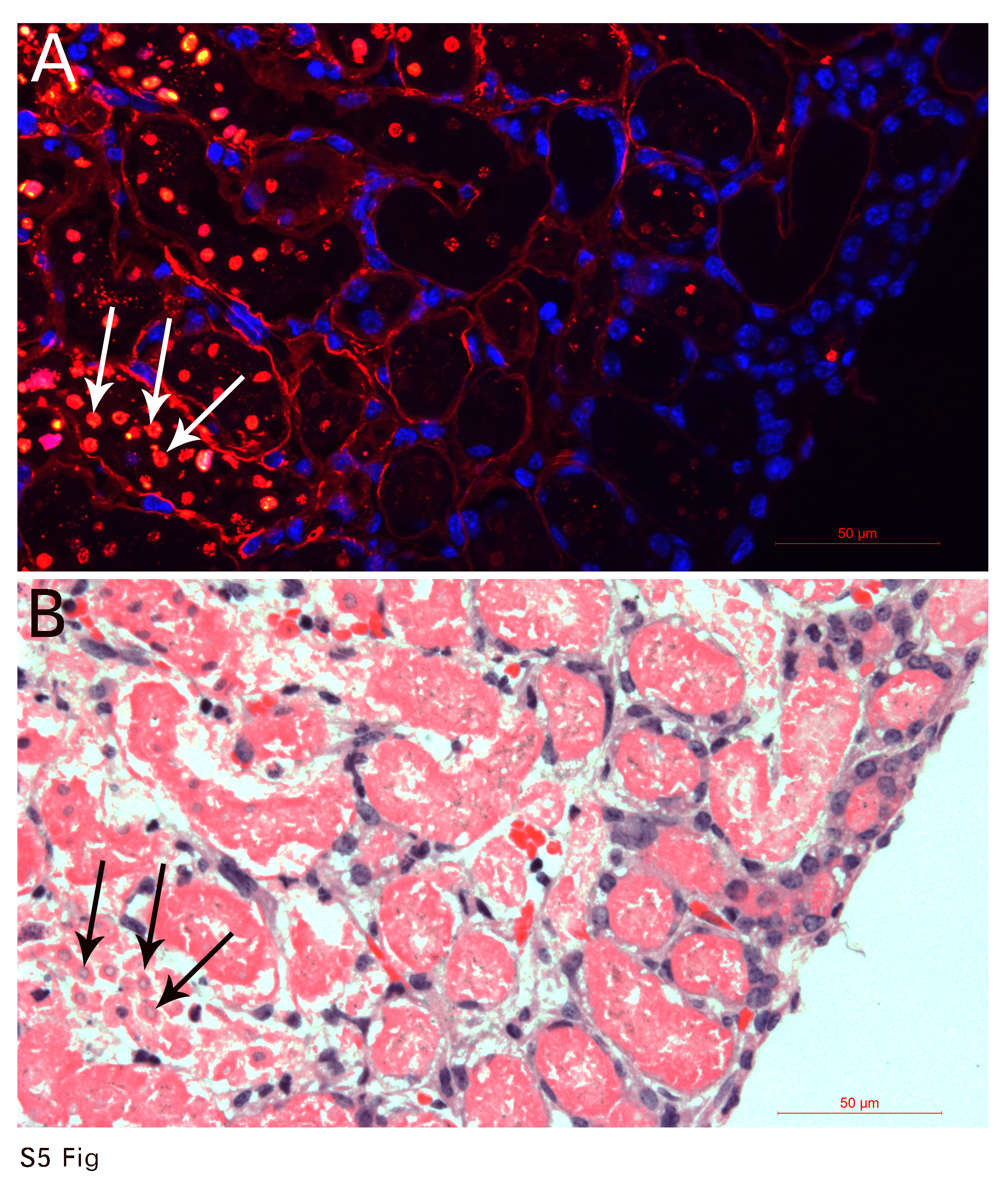

Supplement: S5 Fig — Sections of 1-week ischemia-damaged kidney were stained by TUNEL assay. Nuclei that stained strongly red were apoptotic cells (A). H&E staining of the same section revealed that weak haematoxylin-stained nuclei were apoptotic cells (arrows in A and B). Bars = 50 μm. (TIF) [file pone.0173072.s005.tif]

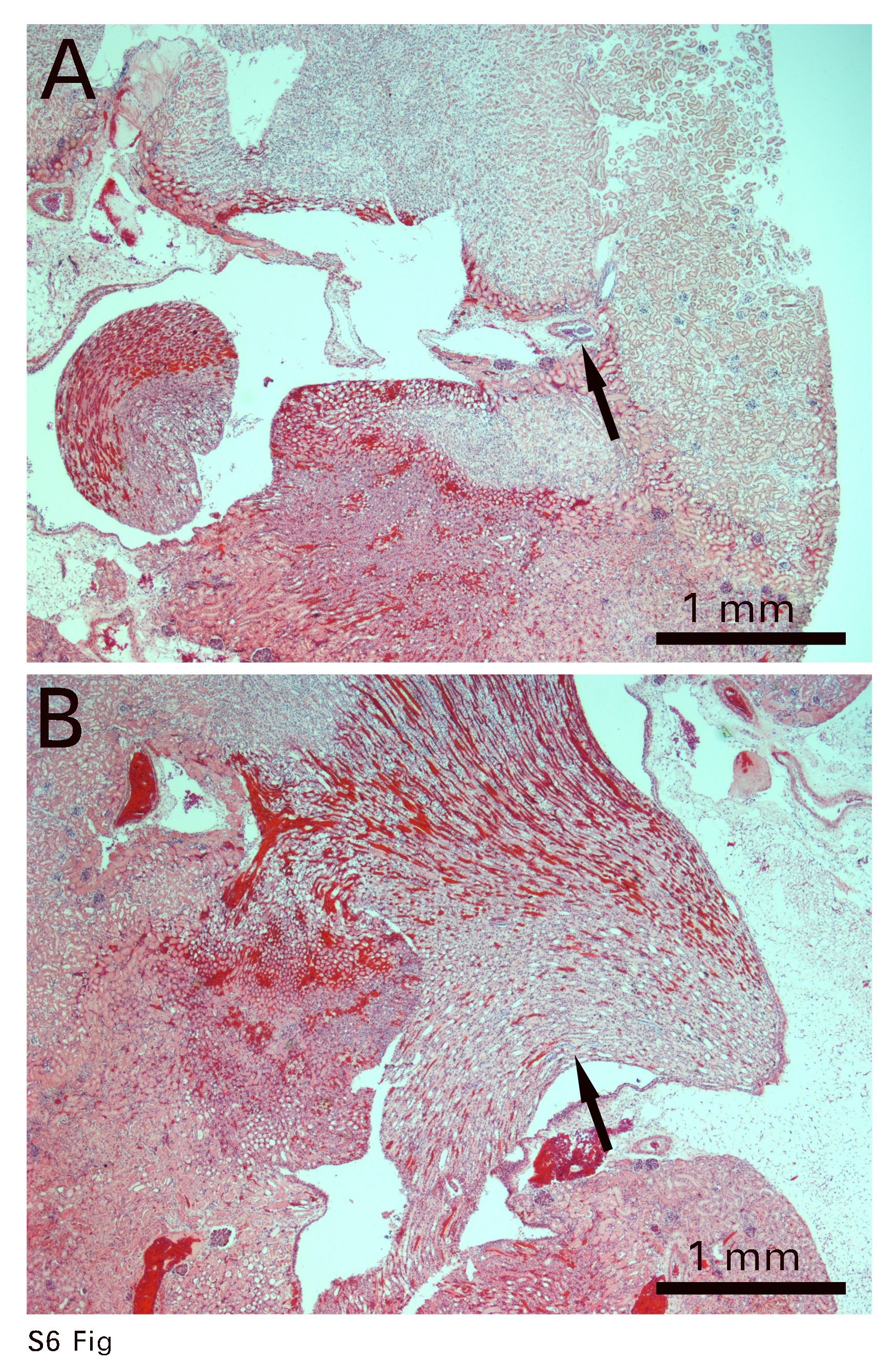

Supplement: S6 Fig — Low magnified images show 2 samples of 1-day ischemia-damaged kidneys. The arrow in A is the blood vessel described in Fig 5. The arrow in B is the grouped sand-like DNA materials described in Fig 6. Bars = 1mm. (TIF) [file pone.0173072.s006.tif]

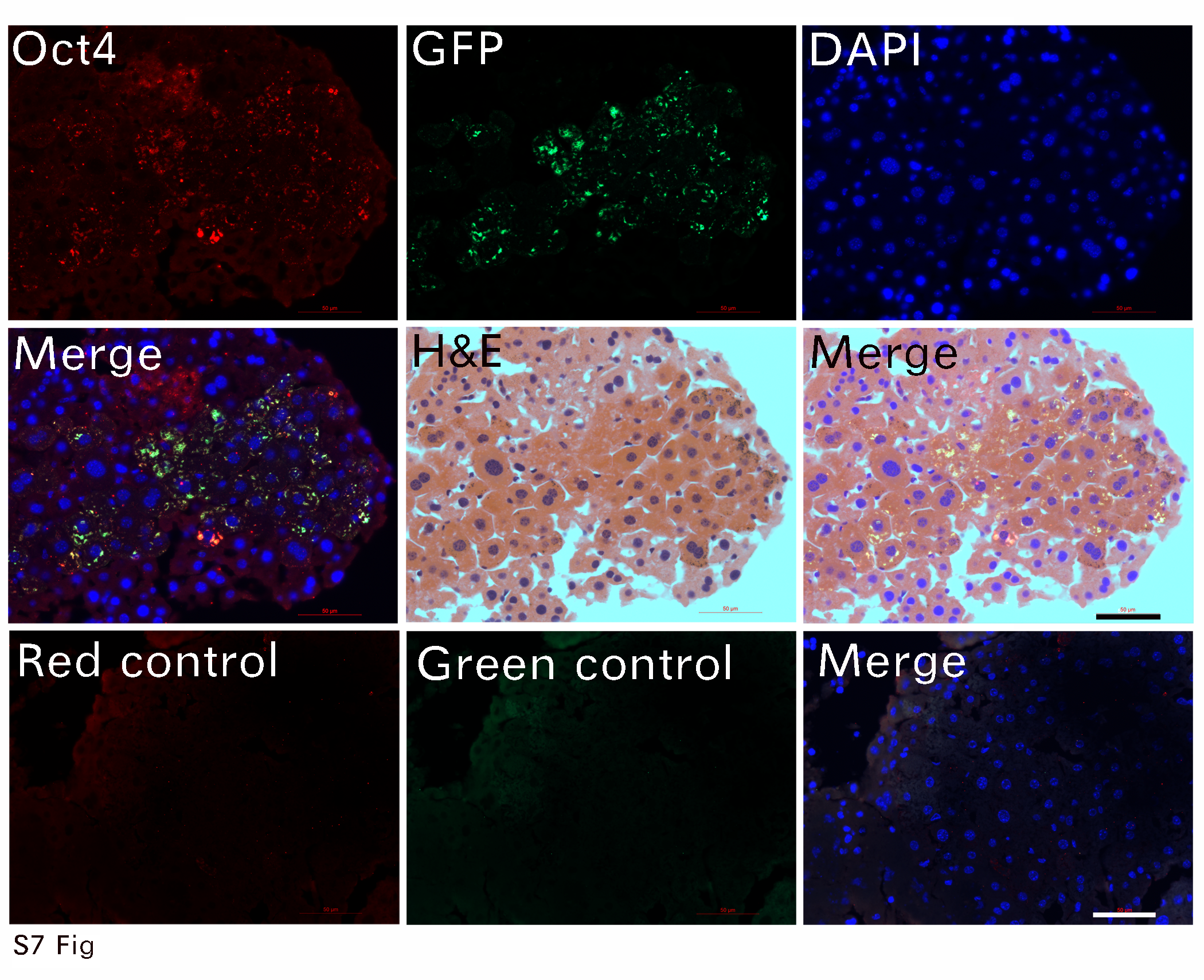

Supplement: S7 Fig — GFP was expressed only in eosin-rich–stained cells. Except for a few cells, OCT4 was also expressed in these cells. Bar = 50 μm. (TIF) [file pone.0173072.s007.tif]

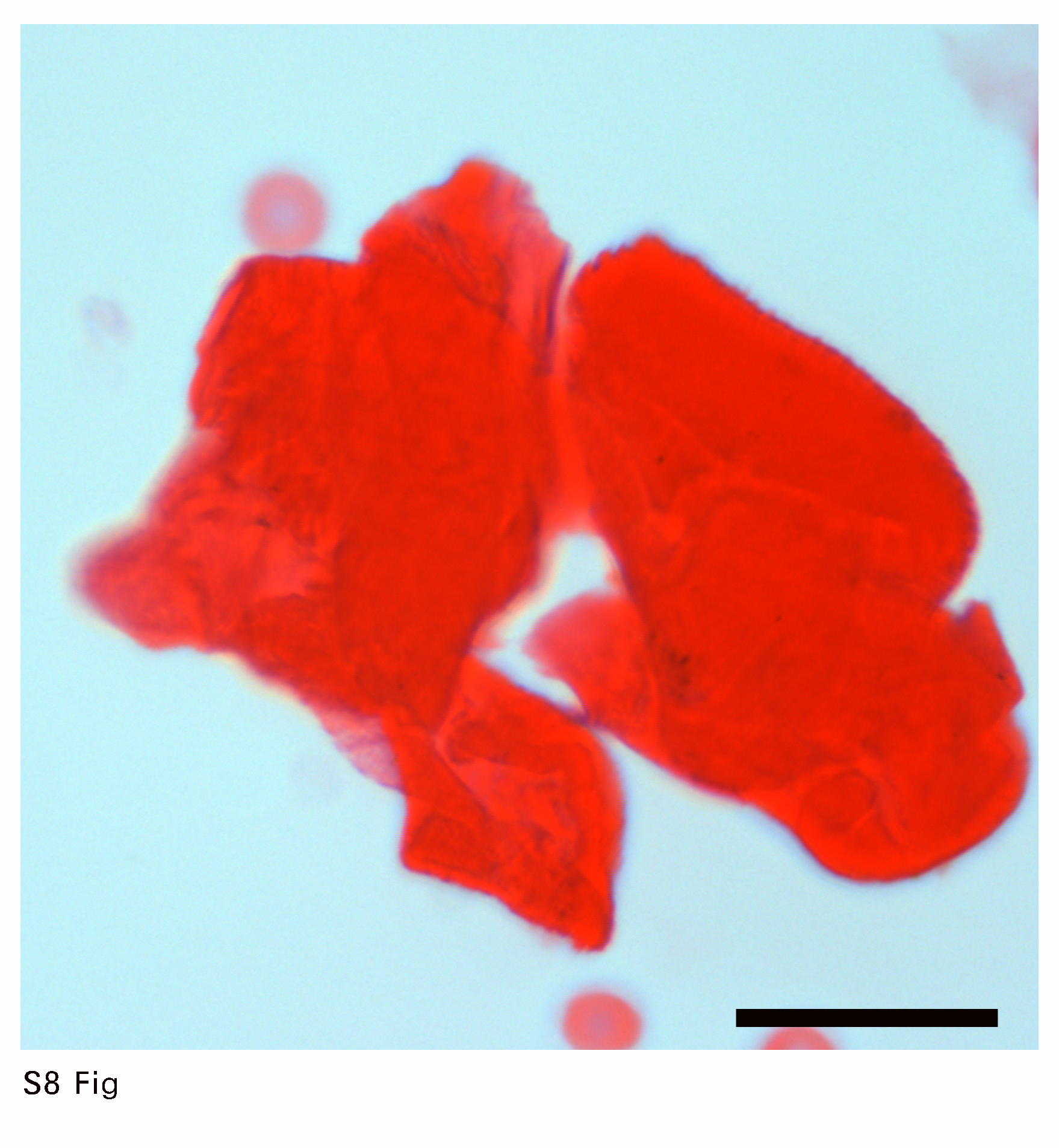

Supplement: S8 Fig — The cellular portion was dropped on slides and stained with H&E. Particle-producing cells were identified and imaged. Bar = 20 μm. (TIF) [file pone.0173072.s008.tif]
